# Quantitative proteomics identifies plasma protein alterations that associate with metabolic and thrombotic profile changes after bariatric surgery

**DOI:** 10.1111/dom.16267

**Published:** 2025-02-25

**Authors:** Hasnain Ahmed, Marco F. Fernandes, Kazim Abbas, Silvia A. Synowsky, Sally L. Shirran, Ramzi A. Ajjan, Alan J. Stewart

**Affiliations:** ^1^ School of Medicine University of St Andrews North Haugh St Andrews Fife UK; ^2^ Renal Transplant Unit Royal Liverpool University Hospital Prescot Street Liverpool Merseyside UK; ^3^ Biomedical Sciences Research Complex and School of Biology University of St Andrews North Haugh St Andrews Fife UK; ^4^ Leeds Institute of Cardiovascular and Metabolic Medicine University of Leeds Clarendon Way Leeds Yorkshire UK

**Keywords:** cardiovascular disease, mass spectrometry, Roux‐en‐Y gastric bypass, SWATH‐MS

## Abstract

**Objective:**

Roux‐en‐Y gastric bypass (RYGB) surgery has been shown to lead to favourable health outcomes in obese patients. However, the molecular changes that occur and how they relate to clinical measures are poorly understood. Here, we characterise the proteomic alterations that occur in plasma of RYGB patients before and 9 months after surgery using quantitative proteomics.

**Methods:**

Plasma proteomics was performed by sequential window acquisition of all theoretical fragment ion spectra mass spectrometry (SWATH‐MS) to identify and quantify differentially abundant proteins. Relationships between significantly altered proteins and clinical markers were examined. A gene set enrichment analysis was also conducted to identify altered pathways.

**Results:**

From the proteomic analysis, 27 proteins increased, and 43 proteins decreased in abundance 9 months after surgery, providing insights into the physiological changes that accompany weight loss. Proteins including sex hormone binding globulin (SHBG), inter‐alpha‐trypsin inhibitor heavy chain 3 (ITIH3) and apolipoprotein D (APOD), which increased in abundance post‐surgery, highlight improvements in lipid regulation, insulin sensitivity and inflammation. Proteins involved in coagulation, including α2‐macroglobulin, kallikrein‐B1, prothrombin, and factor (FX, FXI and FXII), exhibited reduced levels, aligning with a decrease in thrombotic potential.

**Conclusions:**

These findings provide a mechanistic understanding of how bariatric surgery leads to systemic changes in metabolic and haemostatic pathways, thus favourably modulating the risk of developing cardiovascular disease.

## 1 INTRODUCTION

Obesity is a complex condition that significantly elevates the risk of cardiovascular, metabolic and gastrointestinal diseases and various cancers.^1^ Treatment options for obesity include lifestyle modifications, behavioural therapy, pharmacotherapy and bariatric surgery.^2^ Patients with body mass index (BMI) ≥40 kg/m^2^ or those with a BMI between >35 and <40 kg/m^2^ who exhibit comorbid medical conditions frequently qualify for surgical intervention.^3^, ^4^ Surgery is also a viable option for patients where non‐surgical measures have failed to achieve or maintain clinically beneficial weight loss.^4^, ^5^

Laparoscopic Roux‐en‐Y gastric bypass (RYGB) is a surgical procedure that combines restrictive and malabsorptive techniques to promote weight loss.^4^ The procedure involves a reduction in the size of the stomach through the creation of a minor gastric reservoir, facilitating a lower caloric intake,^3^ and decreasing nutrient absorption due to the shortening of the small intestine.^4^ Current observational data has shown that RYGB surgery leads to sustained weight loss in patients and affects hormones that regulate hunger and satiety.^6^, ^7^ Obesity‐related comorbidities, such as diabetes, often improve significantly after surgery, including haemostatic balance restoration and reduced thrombotic risk.^8^, ^9^

Although favourable changes have been observed in RYBG patients, detailed quantitative proteomic analyses focussed on identifying bariatric surgery‐associated protein alterations in plasma and their relationships to clinical measures are lacking. Here, we identify proteomic changes that underpin the beneficial effects of RYGB in plasma taken from patients before and 9 months after RYGB surgery. Quantitative proteomics was performed using sequential window acquisition of all theoretical fragment ion spectra mass spectrometry (SWATH‐MS). Relationships between proteomic changes and anthropomorphic, glycaemic, inflammatory and thrombotic markers were examined.

## 2 MATERIALS AND METHODS

### 2.1 Ethics statement

Blood collection was completed following approval by the National Research Ethics Service Committee Yorkshire and The Humber‐Sheffield (REC reference: 11/H1308/16). Written informed consent was obtained from all participants and protocols were performed as per the Declaration of Helsinki.

### 2.2 Plasma sample collection

Twenty‐five RYGB patients (≥18 years old; 16 females and 9 males) referred for bariatric surgery as a treatment for obesity were recruited from York Hospital, United Kingdom. Participants were excluded if they had an endocrine disorder other than type 2 diabetes, a history of alcohol or drug abuse, significant psychological issues, deep vein thrombosis, anticoagulant use, pregnancy, active malignancy or postoperative complications. Two weeks before surgery patients were placed on an 800–1000 kcal/day diet and commenced taking vitamin and mineral supplements. Blood samples were taken (after ≥6 h fasting) 48 h before surgery and 9 months post‐surgery. Plasma was isolated within 30 min and stored at −80°C until analysis. Anthropomorphic measurements and measurements of metabolic variables, inflammatory and haemostatic markers, including fibrin clot and lysis parameters, were carried out as previously described.^10^, ^11^ Specifically, excess weight was calculated based on an ideal weight corresponding to a BMI of 22 kg/m^2^ for each individual. Furthermore, the use of medications, including antidiabetic medication, statins, calcium channel blockers and ACE inhibitors, remained consistent between the pre‐ and postoperative periods. The comparison of medication use is available in Table S7. Figure S1 shows the density distribution for the anthropomorphic and plasma‐derived clinical parameters stratified by study group (i.e., pre‐ and post‐surgery).

### 2.3 Sample preparation for mass spectrometric (MS) analysis

Citrated plasma containing 50 μg total protein was prepared for MS analysis. For this, plasma proteins were denatured in 5 M urea (Sigma‐Aldrich, Dorset, UK), 2% sodium deoxycholate (Sigma‐Aldrich) and 50 mM ammonium bicarbonate (Sigma‐Aldrich) then reduced and alkylated with 5 mM iodoacetamide (Sigma‐Aldrich) for 45 min. The reaction was quenched with 10 mM dithiothreitol (ThermoFisher Scientific, Paisley, UK) for 30 min and diluted with 50 mM ammonium bicarbonate (Sigma‐Aldrich) to a final urea concentration of 1.5 M. The samples were then digested with 0.2 μg/μl trypsin (Promega, Madison, USA) overnight at 30°C. Finally, 0.5%(v/v) trifluoroacetic acid (TFA) (Sigma‐Aldrich) was added and the samples were centrifuged at 1400 rcf. The peptides were desalted using C18 Micro‐SpinColumns (Harvard Apparatus, Massachusetts, USA). The solvent in each sample was removed using a SpeedVac (ThermoFisher Scientific, Loughborough, UK).

### 2.4 Liquid chromatography‐electrospray ionisation‐tandem mass spectrometry (LC‐ESI‐MSMS) analysis for spectral library generation

The pooled, dried peptides were then resuspended in 100 μL of buffer A, consisting of 10 mM ammonium formate, 2% acetonitrile pH 10.0 (ThermoFisher Scientific). Peptides were then fractionated on a XBridge C18 column (4.6 × 100 mm, 5 μm, Waters) at 1 mL/min with the following gradient: linear gradient of 4–28% buffer B (10 mM ammonium formate, 90% acetonitrile, pH 10.0) for 36 min, then 28–50% B for 8 min, followed by 100% B for 5 min to column wash, before re‐equilibration in 100% buffer A for 10 min. Fractions of 0.5 mL were collected every 30 s. Fractions were pooled to yield 15 fractions across the elution profile, dried, resuspended in 0.1% formic acid (FA) (ThermoFisher Scientific) and then individually analysed on a Fusion Lumos Tribrid MS with a high‐field asymmetric waveform ion mobility spectrometry (FAIMS) interface (ThermoFisher Scientific) coupled to an Ultimate 3000 RSLC (ThermoFisher Scientific) in data‐dependent acquisition (DDA) mode. Additionally, 1 μg of peptides from each individually digested sample for the SWATH‐MS analysis were combined and analysed in DDA mode. Prior to MS analysis, reference iRT peptides (Biognosys, Schlieren, Switzerland) were added to samples to allow correction of retention times. Peptides were injected onto a reverse‐phase trap column (Pepmap100 C18 100 μm × 2 cm) for pre‐concentration and desalted with 0.05% TFA, at 5 μL/min for 10 min. The peptide trap was then switched in‐line with the analytical column (Easy‐Spray Pepmap RSLC C18, 2 μm, 100 A, 75 μm ID × 50 cm). The analytical solvent system consisted of buffer A (100% water, 0.1% FA) and buffer B (80% acetonitrile, 20% water, 0.1% FA) at a flow rate of 300 nL/min, with the following gradient: linear 1–20% of buffer B over 90 min, linear 20–40% of buffer B over 30 min, linear 40–99% of buffer B over 10 min, isocratic 99% of buffer B for 5 min, linear 99–1% of buffer B over 2.5 min and isocratic 1% solvent buffer B for 12.5 min. The FAIMS interface was set to –40 V and –65 V at standard resolution. The MS was operated in DDA positive ion mode with a cycle time of 1.5 s. The Orbitrap was selected as the MS1 detector at a resolution of 120 000 with a scan range of from m/z 375 to 1500; RF‐lens set to 30%. Peptides with charge states of 2–5 at a minimum intensity threshold of 5 × 10^3^ were selected for fragmentation in the Orbitrap using HCD as collision energy. Once selected, a dynamic exclusion for 45 s with a 10‐ppm mass tolerance was applied. A dependent scan was only performed on the single charge state per precursor. The iontrap was selected for data‐dependent MS2 fragmentation with an isolation window of m/z 1.2 and no isolation offset. The peptides were fragmented with fixed HCD as activation type at 28% HCD Collision Energy.

### 2.5 Sequential window acquisition of all theoretical fragment ion spectra mass spectrometry data acquisition

For SWATH‐MS analysis, samples (2 μg) were injected onto the LCMS set‐up as described above with the same gradient and data acquisition was performed in data‐independent acquisition (DIA) mode. The DIA MS method alternates between an MS scan and a tMS2 scan containing 24 scan windows with the FAIMS set to −45 V and − 65 V. For the MS scan, the Orbitrap at 120 000‐resolution was selected as a detector with a m/z range from 400 to 1000. The tMS2 scan uses HCD as activation energy with fragments detected in the Orbitrap at 30 000‐resolution. The first 20 m/z windows are 20 mass units‐wide from 410 to 790 followed by a 30 m/z window from 790 to 820, a 40 m/z window from 820 to 860, a 50 m/z window from 860 to 910 and a 60 m/z window from 910 to 970.

### 2.6 Protein profiles from data‐independent acquisition‐mass spectrometry

Raw files from both DDA and DIA modes were converted to mzML format using ProteoWizard v3.0. A spectral library was generated from pooled DDA data using FragPipe v21.0. The FASTA file employed contained the complete human reference proteome (UniProt accession: UP000005640), plus iRT peptides and sequences added from a universal contaminant library.^12^ Protein identification and quantification were conducted using DIA‐NN v1.8.1 with canonical trypsin/P specificity, allowing for one missed cleavage. Variable modifications were set to N‐terminal methionine excision, carbamidomethylation of cysteine residues and oxidation of methionine. The precursor charge state was restricted to the range of 1–4, with precursor m/z ranging from 300 to 1800 and fragment ions m/z ranging from 200 to 1800. The ‘match‐between‐runs’ option was enabled, and the global false discovery rate (FDR) for precursors was set to <1%. The protein intensities matrix (DIA‐NN output) is available in the Supplementary Information (SI) (supp.xlsx, diannOutput tab). The MS proteomics data have been deposited to the ProteomeXchange Consortium via the PRIDE Partner Repository with the dataset identifier PXD057848.

### 2.7 Differential protein expression analysis

The normalised protein intensities matrix (MaxLFQ algorithm, DIA‐NN output)^13^ was the input for the differential protein expression analysis. Initially, missing matrix values were imputed with a random forest algorithm implemented in R‐missRanger.^14^ The imputed matrix is available in Table S1. The matrix was then log‐transformed using base 2 and quantile normalisation applied. A design matrix was created for the linear model (without intersect) with proteins as the outcome and including patient ID (paired design), surgery (post‐ and pre‐surgery), age and sex in an additive way set as predictors. It fits a linear model and applies empirical Bayes moderation with eBayes implemented in R package‐LIMMA followed by retrieving the top‐ranked differentially expressed features using topTable and adjusting p‐values with the Benjamini–Hochberg (BH) method. Model (LIMMA) estimates are available in Table S2. To aid validation of the proteomics data, an additional set of analyses utilising the hsCRP and fibrinogen protein data (summarised in Table 1), which had been measured using different methods, were performed (Figure S2). MS‐based relative C‐reactive protein abundance measurements were found to associate with hsCRP concentrations across the sample set. Similarly, MS‐based measurement of relative fibrinogen chains α, β and γ (FGA, FGB, FGG) abundances each associated with fibrinogen concentration across the sample set was performed. Note that although PAI‐1 concentrations across the sample set were known, this protein did not appear in the MS analysis. As such a similar comparison with PAI‐1 could not be performed.

**TABLE 1:** Demographic characteristics of the studied population and clinical characteristics including mean anthropometric values, glycaemic and inflammatory markers, haemostatic measures and lipid measures before and 9 months after surgery (*n* = 25). The *p*‐values were calculated using paired Student's *t*‐tests or Wilcoxon matched‐pairs signed rank tests for normally and non‐normally distributed data, respectively.

Characteristics	Values
Age at recruitment (years ± SD)	46 ± 10
% Males (number)	36% (9 out of 25)
% Females (number)	64% (16 out of 25)

	Pre‐surgery	9 months post‐surgery	*p*‐value
Anthropomorphic measures			
Weight (kg ± SD)	149 ± 25	106 ± 20	<0.001
BMI (kg/m^2^ ± SD)	51 ± 7	37 ± 5	<0.001
Waist circumference (WC; cm ± SD)	144 ± 11	118 ± 12	<0.001
Hip circumference (HC; cm ± SD)	136 ± 11	117 ± 11	<0.001
Waist‐to‐hip (WHR) (ratio ± SD)	1.07 ± 0.08	1.01 ± 0.08	0.005
Neck circumference (NC; cm ± SD)	45.8 ± 5.1	40.2 ± 4.5	<0.001
Excess weight (EW) (kg ± SD)	87 ± 22	44 ± 17	<0.001
Visceral fat rating (VFR; % ± SD)	22 ± 7	13 ± 4	<0.001
Glycaemic/inflammatory markers			
HbA1c (% ± SD)	6.5 ± 1.3	5.5 ± 0.5	<0.001
Glucose (mmol/L ± SD)	5.2 ± 1.5	4.5 ± 0.5	0.054
High sensitivity C‐reactive protein (hsCRP; mg/l)	11.8 ± 7.7	2.4 ± 3.6	<0.001
Haemostatic measures			
Fibrinogen (g/L)	2.94 ± 1.21	3.02 ± 1.19	0.810
Plasminogen activator inhibitor −1 (PAI1; pg/mL)	10 560 ± 6681	15 290 ± 6559	0.015
Maximum turbidity (MaxAbs; abs. units)	0.420 ± 0.095	0.305 ± 0.091	<0.001
Time to 50% lysis (s ± SD)	1013 ± 348	971 ± 459	0.717
Time to 100% lysis (s ± SD)	1703 ± 341	1724 ± 362	0.833
Lipid measures			
Total cholesterol (mM ± SD)	5.0 ± 1.1	4.4 ± 0.8	0.033
Triglyceride (mM ± SD)	1.8 ± 0.8	1.6 ± 1.1	0.593
High‐density lipoprotein (HDL; mM ± SD)	1.1 ± 0.2	1.2 ± 0.3	0.144
Low‐density lipoprotein (LDL; mM ± SD)	3.1 ± 1.0	2.7 ± 0.6	0.131
Cholesterol:HDL ratio (ratio ± SD)	4.6 ± 1.1	3.9 ± 0.9	0.009

### 2.8 Linear mixed models (LMM) with interaction terms

To investigate the impact of the interaction effect of surgery (post‐ vs. pre‐surgery) with anthropomorphic and plasma‐derived clinical markers, we utilised an LMM implemented in R lmer package. This model included ‘surgery’, ‘BMI’, ‘age’ and ‘gender’ as fixed effects in an additive way, with an interaction term between ‘surgery’ and each variable of interest (e.g., BMI, HbA1c, weight). A random intercept for ‘patientID’ was incorporated to account for variability between patients. The model can be expressed as ‘protein ~ surgery * VAR + age + gender + (1 | patientID)’. The tilde symbol (~) separates the dependent (response) variable from the independent (predictor) variables. It means “is modelled as a function of”. The asterisk (*) specifies both main effects and their interaction effect. LMM estimates are available in Table S3. For the post hoc analysis, we employed the ‘emmeans’ R package to calculate estimated marginal means and perform pairwise contrasts for the effect of surgery (post‐ vs. pre‐surgery). Raw p‐values were adjusted using the BH method. Estimated coefficients (betas) from the interaction terms of the LMM between surgery (post‐ vs. pre‐surgery) and each anthropometric variable were aggregated into a matrix. Hierarchical clustering was performed on the proteins and the anthropometric variables to identify patterns.

### 2.9 Gene set enrichment analysis (GSEA)

**FIGURE 1:**
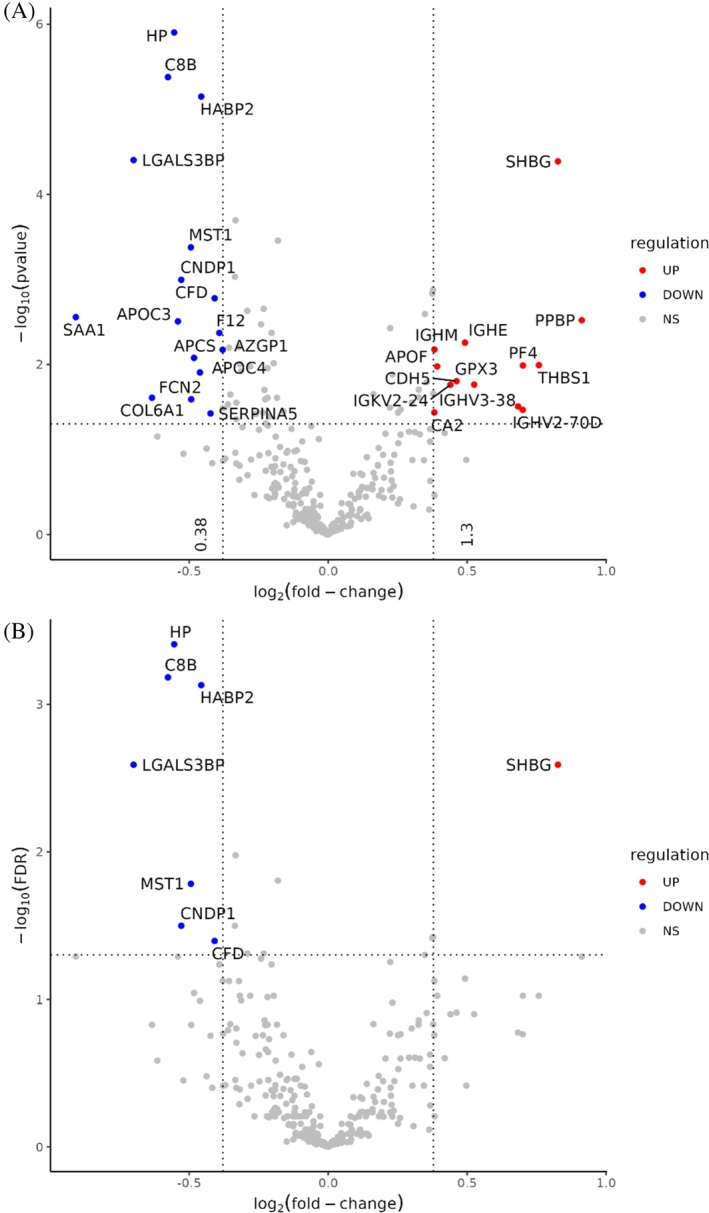
Volcano plots depicting the differential protein expression between the two groups (pre‐surgery and post‐surgery). In each plot, the x‐axis represents the log2‐fold magnitude of change in protein abundance. In A, the y‐axis corresponds to the log10 of the p‐value and in B, the y‐axis corresponds to the –log10 of the FDR. Proteins with a p‐value or FDR <0.05 and a fold‐change greater than 0.3 are highlighted (grey dashed lines). Blue points indicate proteins of lower abundance in the post‐surgery samples, and red points indicate proteins of higher abundance in the post‐surgery group.

Gene set enrichment analysis (GSEA) was performed on proteins exhibiting a significant (*p* < 0.05) increase or decrease in abundance 9 months post‐surgery (compared to pre‐surgery samples) stemming from the linear models made for the volcano plot (Figure 1). Analysis was conducted through the use of the ShinyGO 0.81 (http://bioinformatics.sdstate.edu/go/),^15^ in which subsequent gene identifiers were analysed through the use of pathway databases ‘Curated.Reactome’ stemming from pathways via the Reactome pathway browser (https://reactome.org/).^16^ Parameters were set at FDR cut‐off (<0.05), pathways to show (≤15), pathway size: Min. (≥2) and pathway size: Max. (≤5000). The data are available in Tables S4 and S5.

## 3 RESULTS

### 3.1 Quantitative analysis of proteins from bariatric surgery patients before and 9 months after surgery

From the SWATH‐MS data, 332 unique proteins were identified across the sample‐set. Following intensity distribution analysis, 19 were removed as potential contaminant proteins. The remaining 313 proteins were compared in the samples taken before and 9 months after surgery. Significant differences were observed for 70 proteins between these groups, with 27 proteins increasing in abundance and 43 proteins decreasing in abundance after surgery (Figure 1). Proteins that increased following surgery included sex hormone binding globulin (SHBG), which showed the most significant change (*p* = 4.10 × 10^−5^), followed by inter‐alpha‐trypsin inhibitor heavy chain‐3 (ITIH3; *p* = 1.33 × 10^−3^), apolipoprotein D (APOD; *p* = 1.47 × 10^−3^), alpha‐2‐macroglobulin (A2M; *p* = 2.55 × 10^−3^), pro‐platelet basic protein (PPBP; *p* = 3.02 × 10^−3^) and inter‐alpha‐trypsin inhibitor heavy chain‐1 (ITIH1; *p* = 3.74 × 10^−3^). Proteins that decreased following surgery included haptoglobulin (HP), which showed the most significant change (*p* = 1.25 × 10^−6^), followed by complement component 8B (C8B; *p* = 4.19 × 10^−6^), hyaluronic acid binding protein 2 (HABP2; *p* = 7.10 × 10^−6^), galectin‐3‐binding protein (LGALS3BP; *p* = 3.97 × 10^−5^), kallikrein B1 (KLKB1; *p* = 2.02 × 10^−4^) and prothrombin (F2; *p* = 3.50 × 10^−4^). Multiple hypothesis testing was performed using the BH method. Adjusted p‐values are provided in Table S2.

### 3.2 Relationships between differentially abundant proteins after surgery and patient anthropomorphic and plasma measures

**FIGURE 2:**
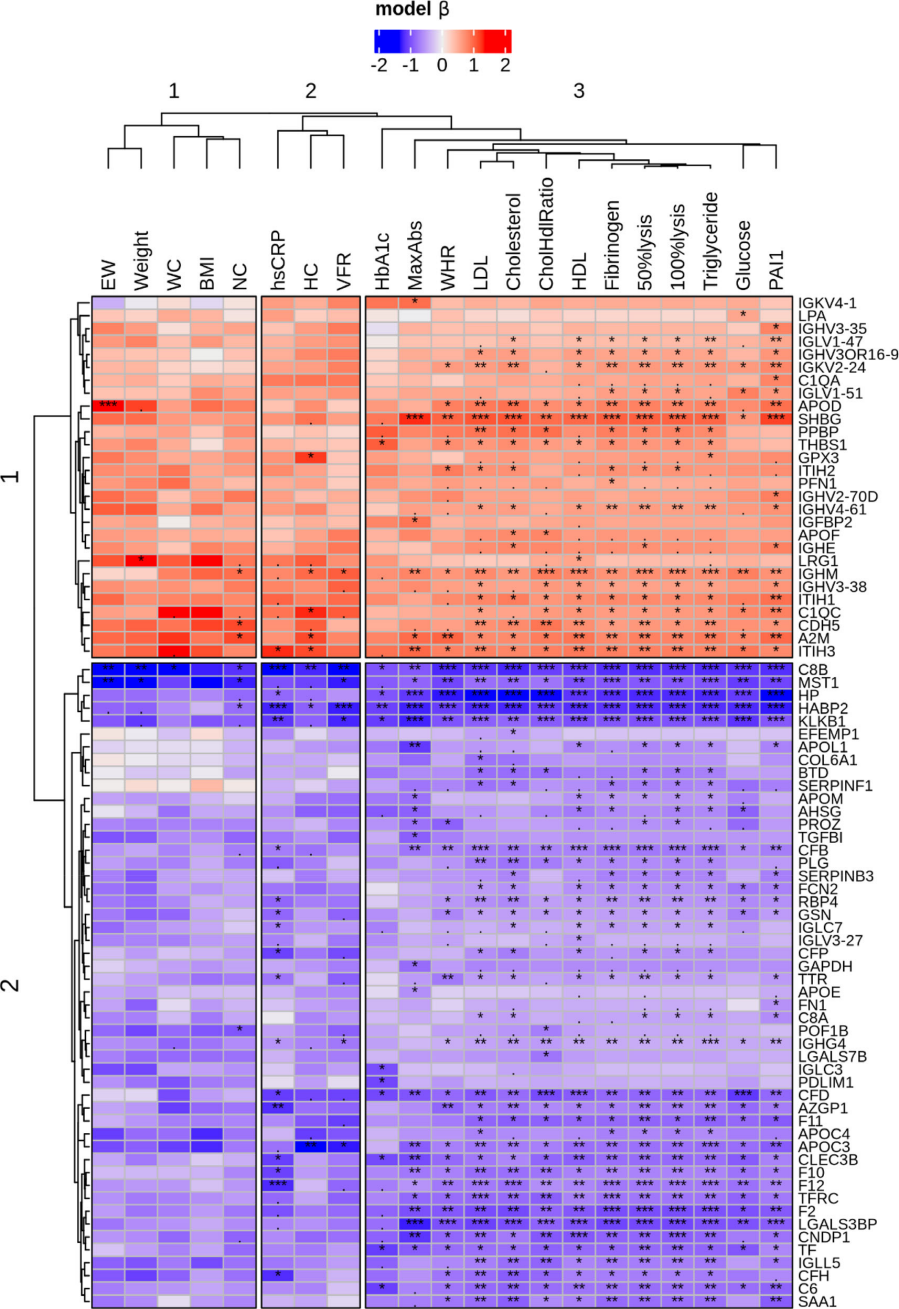
Heatmap with model estimates (betas) from the linear mixed models filtered for proteins with FDR <0.05. Columns represent anthropomorphic and plasma derived clinical parameters. While rows are the gene symbols of proteins. The star annotation represents the FDR (BH) for each level of significance, dot (.) <0.1, one‐star (*) <0.05, two‐stars (**) <0.01 and three‐stars (***) <0.001. Clustering of proteins in rows (k = 2), and anthropomorphic and anthropomorphic and plasma derived parameters in columns (k = 3) are shown.

A pairwise correlation heatmap (Figure 2) visualises relationships between plasma proteins with significantly altered abundance before and 9 months after surgery. The inclusion of clinical measures within this analysis was aimed to highlight potential mechanistic associations with proteins. A summary of the LMM per interaction term is provided in Table S6. The estimates (betas) for each interaction term between the clinical variables and surgery were clustered after filtering for FDR <0.05. Two clusters were observed row‐wise (proteins): cluster‐1 (*n* = 30) with betas >0, and cluster‐2 (*n* = 57) with betas <0. Column‐wise, three clusters are present: cluster‐1 included excess weight (EW), weight, waist circumference (WC), BMI and neck circumference (NC); cluster‐2 contains hip circumference (HC), visceral fat ratio (VFR) and high sensitivity C‐reactive protein (hsCRP) concentration; while cluster‐3 consists of waist to hip ratio (WHR), glucose and HbA1c levels, maximum turbidity (MaxAbs), lysis times (to 50% and 100% lysis), fibrinogen and PAI‐1 levels, cholesterol, LDL, HDL and triglyceride levels and cholesterol to HDL ratio (CholHdlRatio).

Of the higher abundant proteins in the post‐surgery samples, SHBG concentration exhibited significant negative associations with parameters, including weight, VFR, HC, NC, glucose and HbA1c levels, hsCRP concentration, MaxAbs, lysis times (to 50% and 100%), cholesterol and triglyceride concentrations and CholHdlRatio. In addition, proteins that exhibited increased abundance in the post‐surgery samples, including A2M, ITIH3, complement component C1q C chain (C1QC), immunoglobulin heavy constant mu (IGHM), ITIH1, SHBG, PPBP, thrombospondin 1 (THBS1), APOD, cadherin‐5 (CDH5) and glutathione peroxidase 3 (GPX3), showed significant negative correlations with a variety of these measures. APOD positively correlated with fibrinogen concentration. Of the lower abundant proteins post‐surgery, significant positive associations were observed between a group of proteins comprising transferrin receptor protein 1 (TFRC), C‐type lectin domain family 3 member B (CLEC3B), LGALS3BP, HABP2, HP, apolipoprotein C4 (APOC4), serpin family B member 3 (SERPINB3), plasminogen (PLG), zinc‐alpha‐2‐glycoprotein (AZGP1), transferrin (TF), apolipoprotein C3 (APOC3), serum amyloid A1 (SAA1), KLKB1, factor X (F10), F2, complement factor B (CFB), factor XI (F11), factor XII (F12), complement factor H (CFH), C8B, complement component 6 (C6), complement factor D (CFD), macrophage‐stimulating protein (MST1), immunoglobulin lambda‐like polypeptide 5 (IGLL5) and biotinidase (BTD) and parameters including VFR, glucose, HbA1c and hs‐CRP concentrations, MaxAbs, lysis times (50% and 100%), cholesterol and triglyceride concentrations and CholHdlRatio.

### 3.3 Gene set enrichment analysis (GSEA)

**FIGURE 3:**
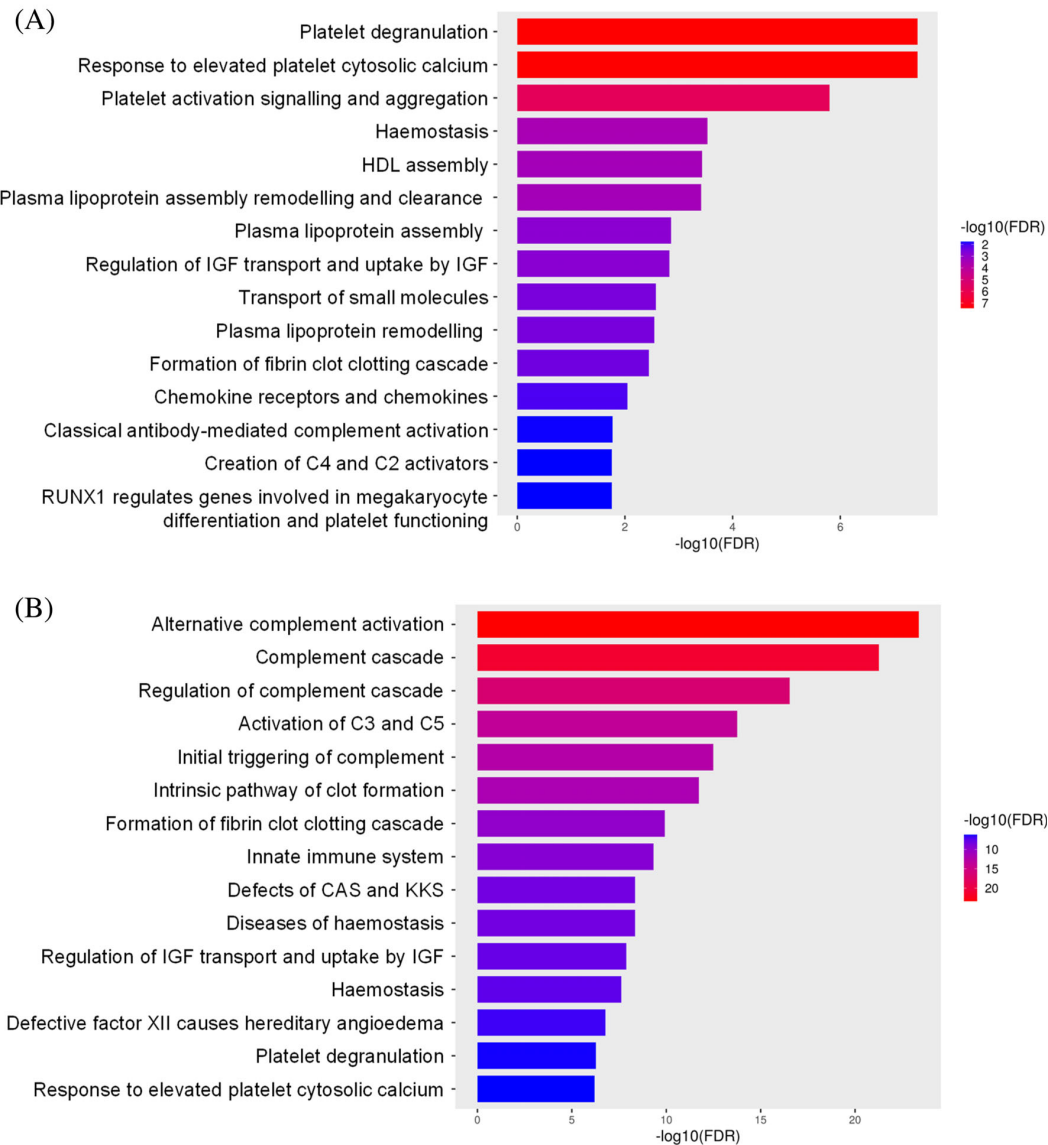
Bar plot stemming from the Curated Reactome pathway gene set enrichment analysis (GSEA) for proteins exhibiting (A) significantly increased abundance 9 months after RYGB surgery compared to pre‐surgery levels and (B) significantly decreased abundance 9 months after RYGB surgery compared to pre‐surgery levels The Y‐axis shows the functional categories, and the X‐axis represents the FDR transformed to –log10. Bars are colour‐coded by the fold enrichment score, ranging from blue to red. A maximum of 15 categories are displayed per analysis. CAS, complement activation system; IGF, insulin‐like growth factor; KKS, kallikrein/kinin system.

An over‐representation analysis, to statistically determine whether proteins from certain pathways are enriched in the data, was performed on proteins displaying differential abundance pre‐surgery and at 9 months post‐surgery using the Reactome pathway using ShineyGO (Figure 3). Proteins that are expressed at higher levels in patients after surgery are enriched in biological processes related to platelet function‐associated pathways, ‘haemostasis’ and ‘Plasma lipoprotein assembly’. Proteins that are of lower abundance in patients within the 9 months post‐surgery group are involved in complement and haemostasis‐related pathways.

## 4 DISCUSSION

This study utilised SWATH‐MS to track plasma proteomic changes in obese patients 9 months after RYGB, offering insights into molecular changes following significant weight loss. This approach has also been used in other studies, such as that by Iqbal et al. who explored protein changes linked to type 2 diabetes remission after bariatric surgery, observing significant shifts in proteins like SHBG, TF and apolipoprotein A4 (APOA4).^17^ Our study focuses on proteomic alterations after 9 months, a point at which excess weight loss generally stabilises,^18^ and examines relationships with clinical measures, including fibrin clot parameter data,^10^ enabling insights into the improved haemostatic profile after surgery.

In the current study, 27 plasma proteins increased, and 43 proteins decreased in concentration 9 months after surgery. Among the increased proteins, SHBG, a circulating glycoprotein that binds sex hormones and regulates their bioavailability,^19^ showed the most significant change. An increase in SHBG levels has been shown to be associated with improved insulin sensitivity.^20^ In agreement with the study by Iqbal et al.,^17^ SHBG exhibited the highest fold‐change increase post‐surgery. Other increased proteins include ITIH3, APOD, A2M and PPBP. ITIH3 is linked to tissue remodelling, and insulin resistance.^21^ APOD is involved in lipid trafficking, dietary intake regulation, inflammation, antioxidant response and has been shown to stimulate carcinogenesis.^22^ A2M contributes to haemostasis by inhibiting haemostatic enzymes such as thrombin, factor Xa, activated, plasmin, tissue plasminogen activator and urokinase.^23^ PPBP, the precursor to platelet basic protein (PBP), functions as a chemoattractant and activator of neutrophils.^24^ These proteins, among others that increased like apolipoprotein A1 (APOA1), apolipoprotein B (APOB), apolipoprotein f (APOF), APOD, lecithin–cholesterol acyltransferase (LCAT), APOA4, lipoprotein(a) (LPA) and insulin‐like growth factor binding protein 2 (IGFBP2), highlight positive metabolic and coagulative shifts post‐surgery, reflecting broader effects on lipid metabolism and transport and haemostasis.

Of the decreased proteins, HP showed the most significant reduction (*p* = 1.25 × 10^−6^). HP is secreted into blood plasma where it binds haemoglobin, playing a role in tissue protection from haemoglobin‐derived oxidative damage.^25^ HP levels increase during states of inflammation, infection and malignancy and are linked with conditions such as diabetes, heart failure, myocardial infarction and stroke.^26^ Other decreased proteins include C8B, HAPB2 and KLKB1. C8B is involved in formation of the membrane attack complex within the complement cascade.^27^ HAPB2 is an extracellular serine protease which plays vital roles in both the extrinsic pathway of coagulation and fibrinolysis,^28^ and has been linked to various cardiovascular diseases.^28^, ^29^, ^30^, ^31^ Kallikrein B1 is a glycoprotein involved in the surface‐dependant activation of blood coagulation as well as aiding in the regulation of blood pressure and inflammatory processes.^32^ KLKB1 also converts Factor XII (FXII) to FXIIa, which subsequently activates Factor XI (FXI), which contributes to the intrinsic coagulation pathway, leading to thrombin generation and fibrin clot formation.^33^

Several other proteins involved in the coagulation cascade were significantly reduced 9 months after surgery. These included F10, F11, F12, PLG, protein S (PROS1), heparin cofactor II (SERPIND1), protein c inhibitor (SERPINA5) and protein Z (PROZ), with each playing key roles in blood clot formation, wound healing and maintenance of vascular integrity. F2 and PLG play essential roles in the coagulation process, with F2 being the precursor to thrombin facilitating clot formation via fibrin formation.^33^ Upregulation of F2 results in excess thrombin generation and subsequent fibrin network formation, thus increasing the risk of thrombotic events.^34^ This aligns with our previous study, where significantly denser clots were observed in the pre‐surgery group,^10^ highlighting a shift towards forming less dense clots, possibly reducing thrombotic risk. An increased tendency to clot formation pre‐surgery was also evident in the higher levels of FX, FXI and FXII compared with post‐surgery. The reduction in PLG levels post‐surgery may be related to decreased activation of the coagulation system and therefore a lower need for an active fibrinolytic system.

Significant associations between protein levels and clinical markers highlight the impact of bariatric surgery on health, underscoring links between protein expression and clinical measures pre‐and post‐surgery. Within the group of proteins that significantly increased in abundance post‐surgery, SHBG demonstrated negative associations with various anthropomorphic measures, all clot parameters and cholesterol, triglyceride within the post‐group. A cluster of proteins including A2M, ITIH3, C1QC, IGHM, ITIH1, SHBG, PPBP, THBS1, APOD, CDH5 and GPX3 also negatively correlated with several measures. These relationships suggest these proteins play important roles in metabolic, immune and haemostatic pathways. Gene ontology analysis revealed that proteins involved in haemostasis regulation and lipid‐protein assembly were prominently upregulated post‐surgery.

Proteins that decreased in abundance, including TFRC, HABP2, HP, APOC4, KLKB1, PLG, F10, F2, CFB, F11, F12, CFH, C6, C8B, MST1 and CFD, were positively associated with VFR, glycaemic markers, hs‐CRP concentration, all clot parameters and cholesterol and triglyceride concentrations. CFD, MST1 and C8B, which had particularly low false discovery rates, have not been shown previously to differ in abundance following gastric bypass surgery. Gene ontology analysis revealed that proteins involved in complement pathway activation were downregulated post‐surgery. Reduction in the levels of some complement proteins is known to correlate with improvements in glycaemia after bariatric surgery.^35^, ^36^ The decrease in complement proteins we observed after surgery may also reflect reduced systemic inflammation and improvement in liver or endothelial functioning.^37^, ^38^ In addition, many downregulated proteins are involved in the coagulation cascade and likely contribute to the favourable changes in fibrin clot formation observed after surgery.^10^

Limitations of this study involve the size of the participant cohort (*n* = 25). A larger sample size would enable more robust identification of differentially abundant proteins and improve statistical power, thereby enhancing the findings. Despite this, the current study to our knowledge represents one of the largest cohorts directly comparing pre‐ and post‐gastric bypass surgery patients, highlighting its significance within this research context. Additional limitations include the absence of depletion of highly abundant proteins, which can hinder the detection of lower‐abundance proteins. Abundant proteins comprise over 90% of total protein content. Depleting these abundant proteins can allow the detection of low‐abundance targets and reduce the complexity of plasma samples. Although depletion is advantageous in uncovering potentially masked proteins, the Iqbal et al. study,^17^ which utilised immunodepletion of the top 12 abundant proteins using depletion spin columns, identified some of the same differentially abundant proteins reported here, including SHBG, ITIH3, TF, A2M, LGALS3BP and HP. Moreover, depletion has also been known to cause bias due to inadvertently altering sample composition, compromising data quality, reproducibility and data interpretation.^39^ In addition, our proteomic analysis was conducted on samples collected in a fasting state to minimise variability which can be introduced by dietary intake, which will differ between participants, hence, ensuring better comparability between individuals. However, it is important to note that fasting can induce changes in the proteome.^40^ Future studies incorporating both fasting and postprandial analyses could further elucidate the mechanisms underlying RYGB‐associated metabolic changes.

## 5 CONCLUSION

SWATH‐MS identified significant changes in the plasma proteome 9 months after bariatric surgery (compared to pre‐surgery levels). From the results, 27 proteins increased in abundance after surgery and 43 proteins decreased (*p* < 0.05), providing insights into the physiological shifts accompanying weight loss. Specific changes in the abundance of proteins related to lipid metabolism, haemostasis, complement activation and immune response were observed. Proteins such as SHBG, ITIH3 and APOD increased in abundance post‐surgery, highlighting the improvement in lipid regulation, insulin sensitivity and potential anti‐inflammatory effects. These findings coincide with current literature regarding obesity. Proteins involved in the coagulation cascade, including A2M, KLKB1 and coagulation factors F10, F11 and F12, exhibited reduced levels, which align with a decreased thrombotic profile and the formation of less dense fibrin clots. These findings suggest bariatric surgery induces systemic changes in metabolic and haemostatic pathways, alongside weight loss, potentially lowering cardiovascular risk. In conclusion, this work highlights the benefits of bariatric surgery to improve health and offers a better understanding of the mechanisms underlying associated health improvements.

## AUTHOR CONTRIBUTIONS

H.A., R.A.A. and A.J.S.: conceptualisation; K.A.: collection of samples; H.A., S.A.S. and S.L.S.: collection of data; H.A., M.F.F., S.A.S. and S.L.S.: data analysis; M.F.F.: data curation; H.A., M.F.F., A.J.S.: drafting the manuscript; all authors contributed to reviewing and editing the manuscript.

## CONFLICT OF INTEREST STATEMENT

The authors declare no conflicts of interest.

### PEER REVIEW

The peer review history for this article is available at https://www.webofscience.com/api/gateway/wos/peer‐review/10.1111/dom.16267.

## Supporting information


**Figure S1.** Density distribution for the anthropomorphic and plasma derived clinical parameters stratified by bariatric surgery.
**Figure S2.** ELISA and Mass Spectrometry (MS) data comparison for selected plasma proteins.

**Table S1.** Normalised protein intensities matrix.
**Table S2.** Linear model with paired‐design for post‐ and pre‐surgery.
**Table S3.** Linear mixed model to investigate the impact of the interaction effect of surgery (post‐ vs pre‐surgery) with anthropomorphic and plasma‐derived clinical markers.
**Table S4.** Gene set enrichment analysis of plasma protein that increase in abundance 9 months after surgery.
**Table S5.** Gene set enrichment analysis of plasma protein that decrease in abundance 9 months after surgery.
**Table S6.** Summary of linear mixed model per interaction term data.
**Table S7.** Overview of the difference in medication use in patients pre‐surgery and post‐surgery.

## Data Availability

The MS proteomics data have been deposited to the ProteomeXchange Consortium via the PRIDE Partner Repository with the dataset identifier PXD057848.
