# Supplementary material for: Quantitative proteomics identifies plasma protein alterations that associate with metabolic and thrombotic profile changes after bariatric surgery
Source: Diabetes Obes Metab. 2025 Feb 25;27(5):2647–57. doi: 10.1111/dom.16267 (PMC11965011; doi:10.1111/dom.16267)
Supplement: Supplementary file 1 — Figure S1. Density distribution for the anthropomorphic and plasma derived clinical parameters stratified by bariatric surgery. Figure S2. ELISA and Mass Spectrometry (MS) data comparison for selected plasma proteins. [file DOM-27-2647-s001.docx]

**Quantitative proteomics identifies plasma protein alterations that associate with metabolic and thrombotic profile changes after bariatric surgery.**

Hasnain Ahmed, Marco F. Fernandes, Kazim Abbas, Silvia A. Synowsky, Sally L. Shirran, Ramzi. A. Ajjan and Alan. J. Stewart

**Supplementary Information**

**Figure S1. Density distribution for the anthropomorphic and plasma derived clinical parameters stratified by bariatric surgery.**

**Figure S2. ELISA and Mass Spectrometry (MS) data comparison for selected plasma proteins**.


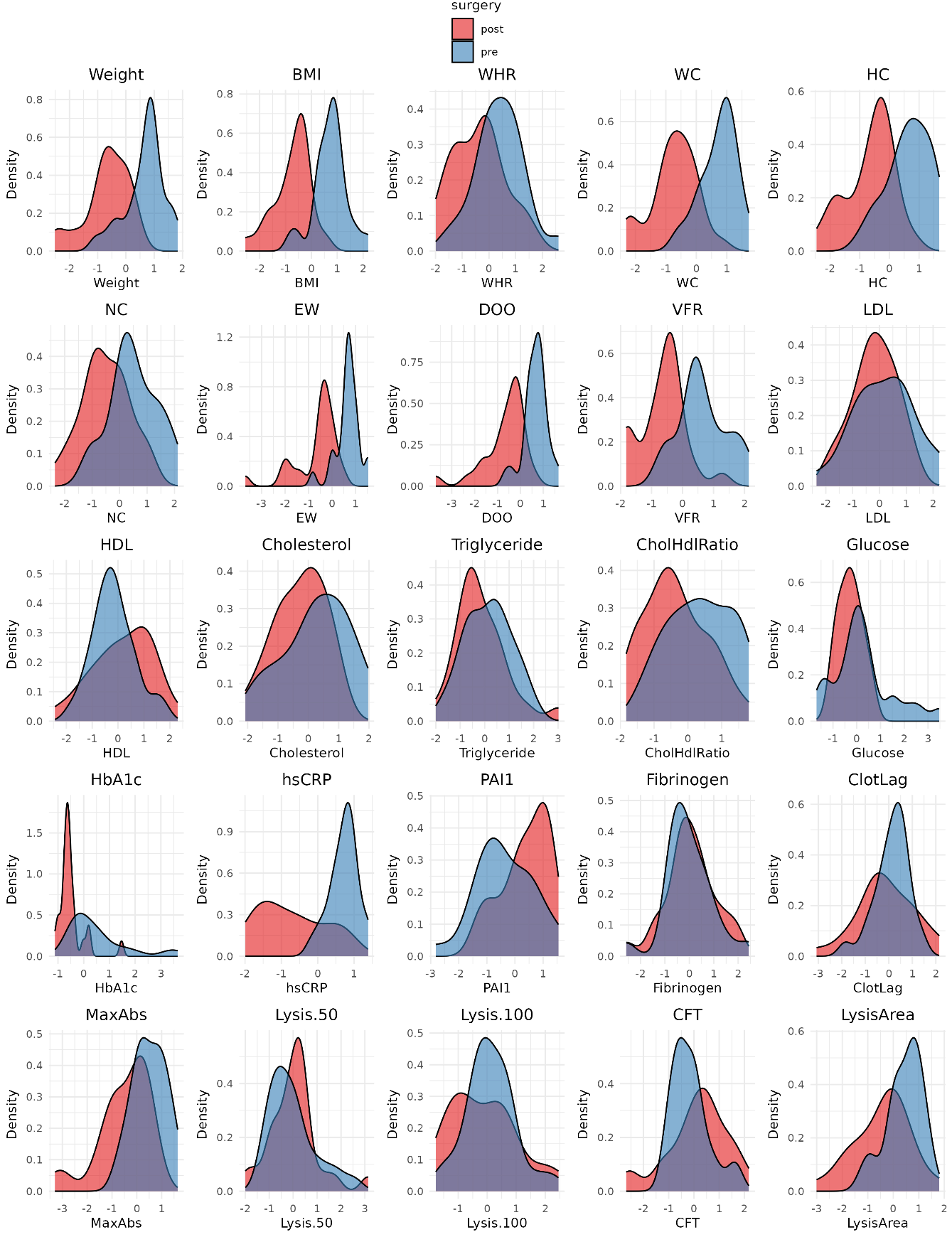


**Figure S1 Density distribution for the anthropomorphic and plasma derived clinical parameters stratified by bariatric surgery**. Each parameter was Log_10_ transformed followed by Z-score normalization.


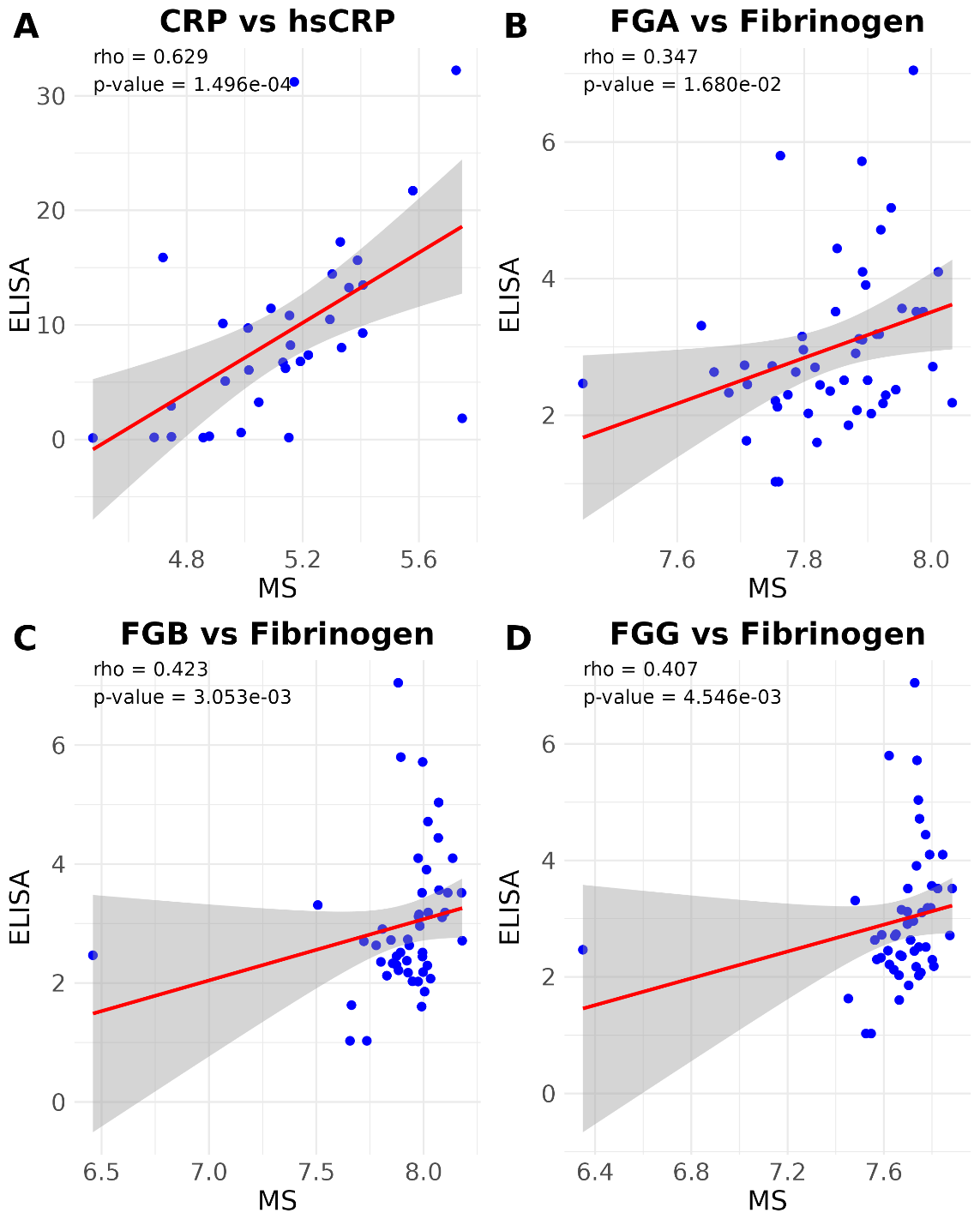


**Figure S2** **ELISA and Mass Spectrometry (MS) data comparison for selected plasma proteins**. C-reactive protein (CRP, **A**), Fibrinogen alpha chain (FGA, **B**), Fibrinogen beta chain (FBB, **C**) Fibrinogen gamma chain (FGG, **D**). Spearman Rank correlation (*rho* coefficient) and associated *p*-value. Linear fit (red line) and confidence interval (CI) at 95% (grey fill). ELISA and MS values are on the Log_10_ scale.
